# Supplementary material for: Ultrafast energy quenching mechanism of LHCSR3-dependent photoprotection in Chlamydomonas
Source: Nat Commun. 2024 May 24;15:4437. doi: 10.1038/s41467-024-48789-x (PMC11126702; doi:10.1038/s41467-024-48789-x)
Supplement: Supplementary file 1 — Supplementary Information [file 41467_2024_48789_MOESM1_ESM.pdf]

Supporting Information for

Ultrafast energy quenching mechanism of LHCSR3-dependent photoprotection in *Chlamydomonas*

Mengyuan Zheng[1, 2, 3 ‡], Xiaojie Pang[1, 2, 3 ‡], Ming Chen[1, 2], and Lijin Tian[1, 2, 3 *]

[1]Key Laboratory of Photobiology, Institute of Botany, Chinese Academy of Sciences, Beijing, 100093, China.

[2]China National Botanical Garden, Beijing, 100093, China.

[3]University of Chinese Academy of Sciences, Beijing, 100049, China.

Corresponding author: Lijin Tian, Email: ltian@ibcas.ac.cn

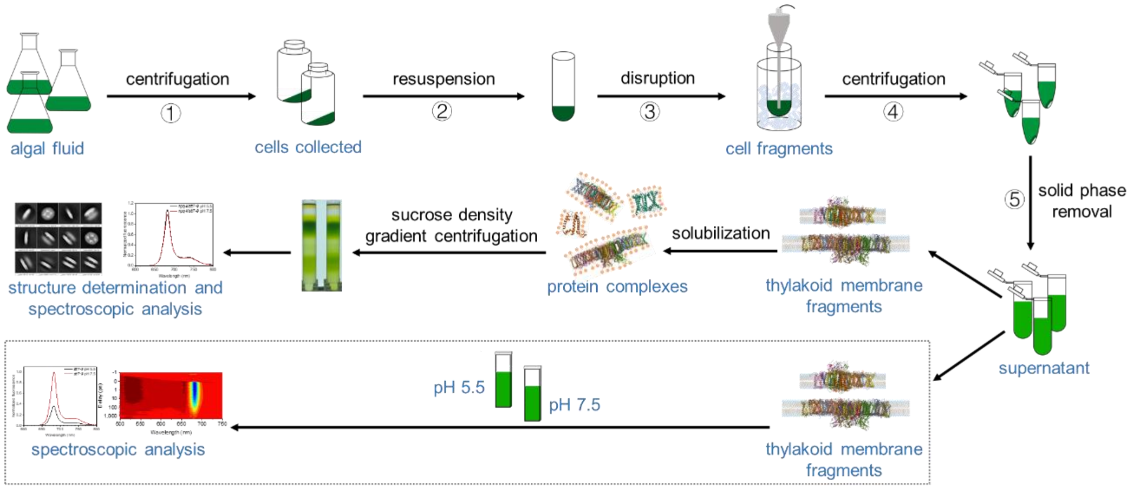

Supplementary Fig. 1. Schematic diagram of the often-used extraction protocol of membrane proteins versus our protocol for the preparation of micro-sized cell fractions. The latter one mainly consists of five steps (①-⑤), and the obtained supernatant was directly used in our spectroscopic studies, see the doted box, instead, for protein 3D structure determination or functional studies in previous publications, two additional steps, the solubilization and sucrose gradient centrifugation, had been further applied.

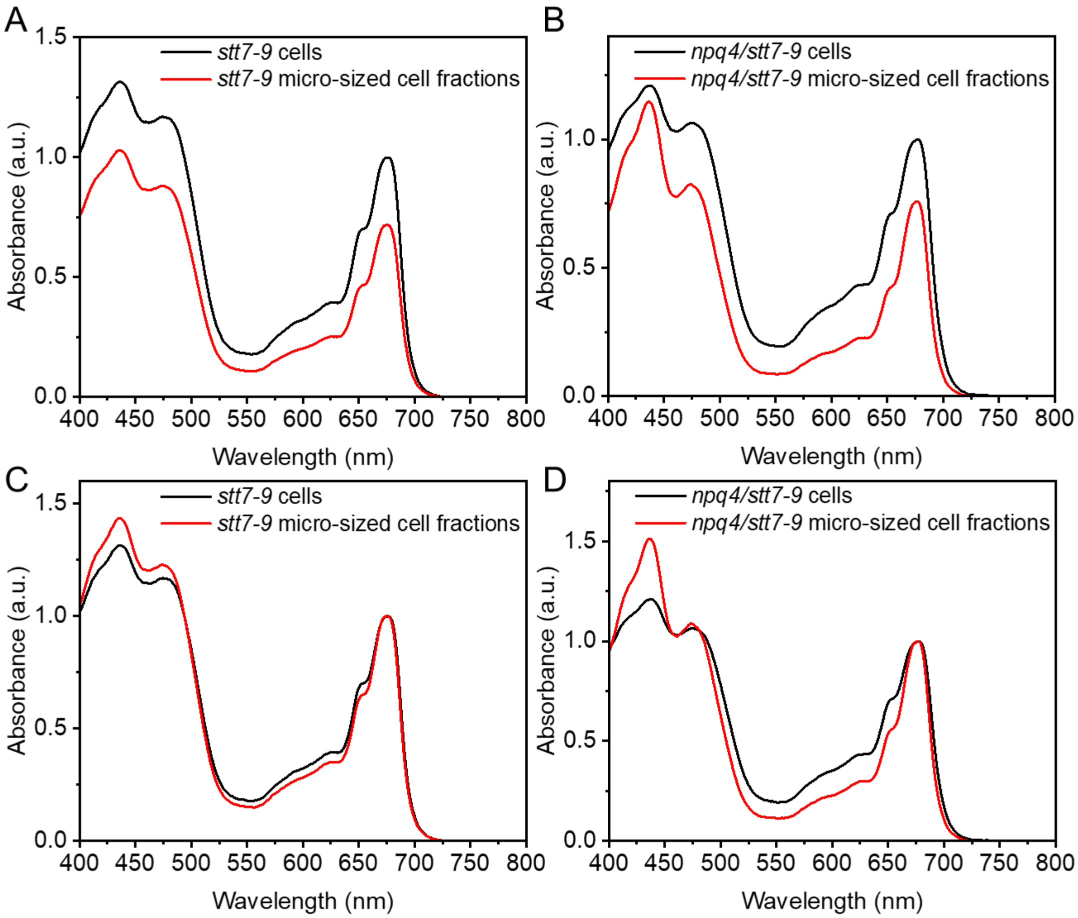

Supplementary Fig. 2. Room temperature absorption spectra before and after cell disruption. All spectra were recorded by using an integrating sphere. The (A) and (B) respectively represent the absorption spectrum of cells (black) and lysed cell fractions (red) of *stt7-9* and *npq4/stt7-9* at pH 5.5. The spectra in (A) and (B) were normalized relatively to the absorption of intact cells at 676 nm to highlight the loss of photosynthetic proteins during preparation. The spectra in (C) and (D) were normalized at 676 nm to show that there is no blue shift in the absorption spectra that is usually led by free pigments or detached antenna. Source data are provided as a Source Data file.

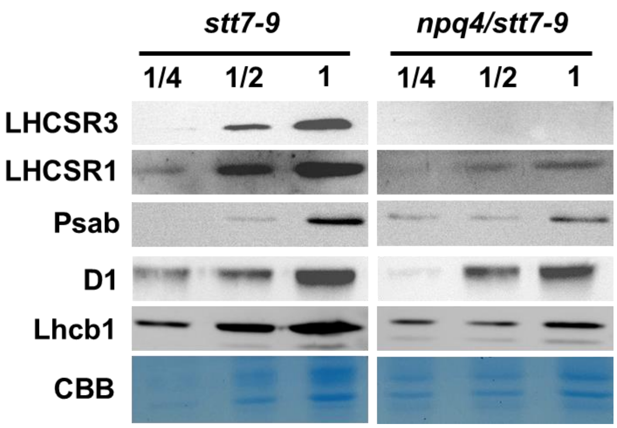

Supplementary Fig. 3. Western blot of micro-sized cell fractions of *stt7-9* (left panel) and *npq4/stt7-9* (right panel). After blotting, the blots were incubated with LHCSR3, LHSCR1, Psab, D1, and Lhcb1 antibodies, respectively. For each sample, a dilution series (1/4, 1/2, and 1 corresponding to 2.5 µg, 5 µg, and 10 µg total protein, respectively) of the protein sample was loaded. CBB refers to the Coomassie blue strained gel image. Note that the *stt7-9* sample contains more LHCSR3 than LHCSR1 as the binding affinity of LHCSR3-antibody to LHCSR3 is much weaker than the one for LHCSR1[1]. The experiment was performed in triplicate. Source data are provided as a Source Data file.

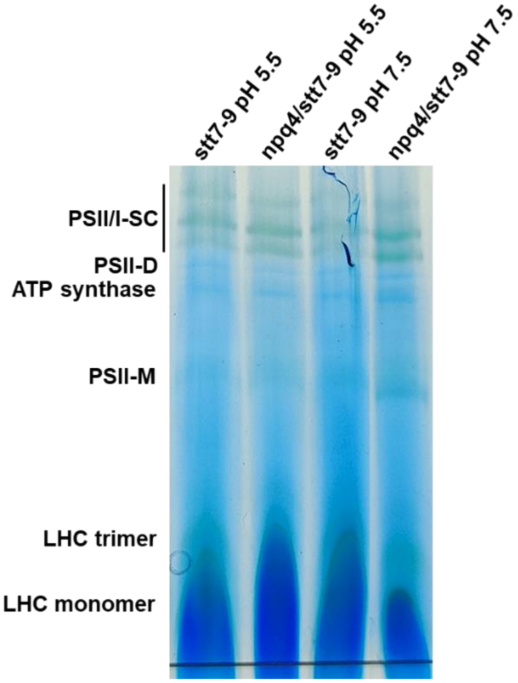

Supplementary Fig. 4. BN-PAGE analyses of micro-sized cell fractions of *stt7-9* and *npq4/stt7-9* at two different pHs. Identification of the thylakoid membrane protein complexes was made according to the previous report[2]. PSII/I-SC: PSII-LHCII super-complex and PSI-LHCI super-complex; PSII-D, PSII dimer; PSII-M, PSII monomer. The experiment was performed in triplicate. Source data are provided as a Source Data file.

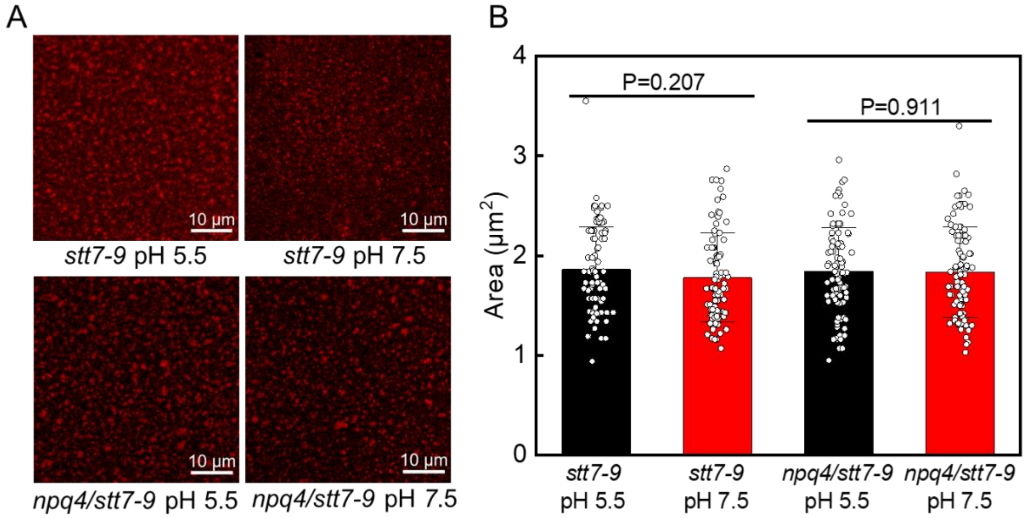

Supplementary Fig. 5. Fluorescence confocal microscopy images and estimated area of the two samples at different pHs. (A) The upper panels represent the images of *stt7-9* samples, and the lower panels show the images of *npq4/stt7-9* samples. Scale bar, 10 μm. (B) The area of samples was calculated from (A). The dark and red bars represent the area of the sample at pH 5.5 and pH 7.5, respectively. Error bar, SD (n=95) (with one-way ANOVA test performed). The area was calculated using the Image J software. The experiment was performed in triplicate. Source data are provided as a Source Data file.

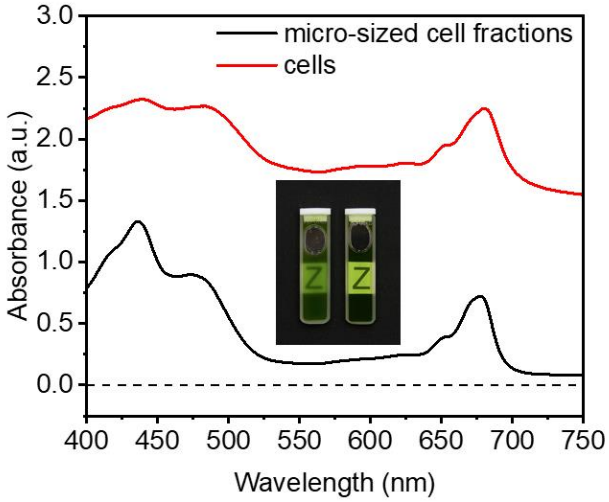

Supplementary Fig. 6. Absorption spectra of the micro-sized cell fractions and *stt7-9* cells at the same chlorophyll concentration. Both were measured without using an integrating sphere. Inset: The images of cells (left) and micro-sized cell fractions (right) show that the former is less transparent because of its high optical scattering. Source data are provided as a Source Data file.

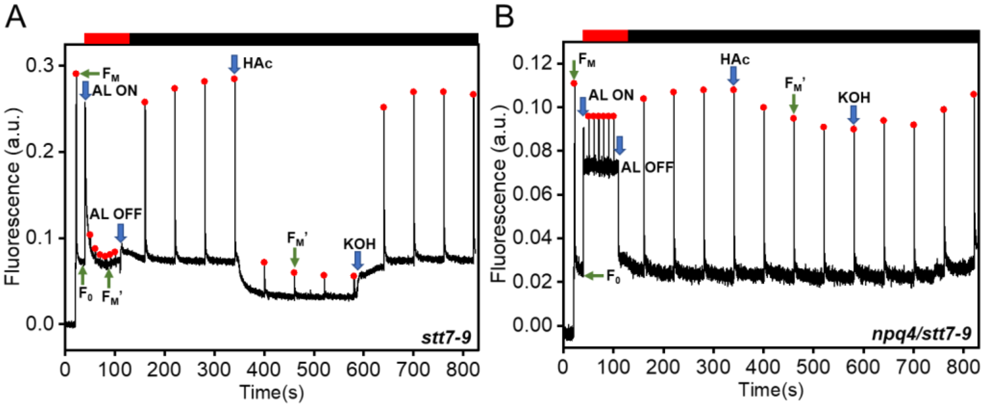

Supplementary Fig. 7. The typical PAM Chl fluorometer traces of steady-state fluorescence in *stt7-9* and *npq4/stt7-9*. (A) the qE of *stt7-9* was induced with actinic light (AL, 40 to 110 s), as well as by adding acetic acid (5 mM, pH 5.5) (340 to 580 s). The quenching was fully reversible after KOH addition setting the pH back to 7.5 (580 to 820 s). (B) The same chlorophyll fluorescence measurement process was applied to *npq4/stt7-9*. The red dots indicate F_M and F_M'. Source data are provided as a Source Data file.

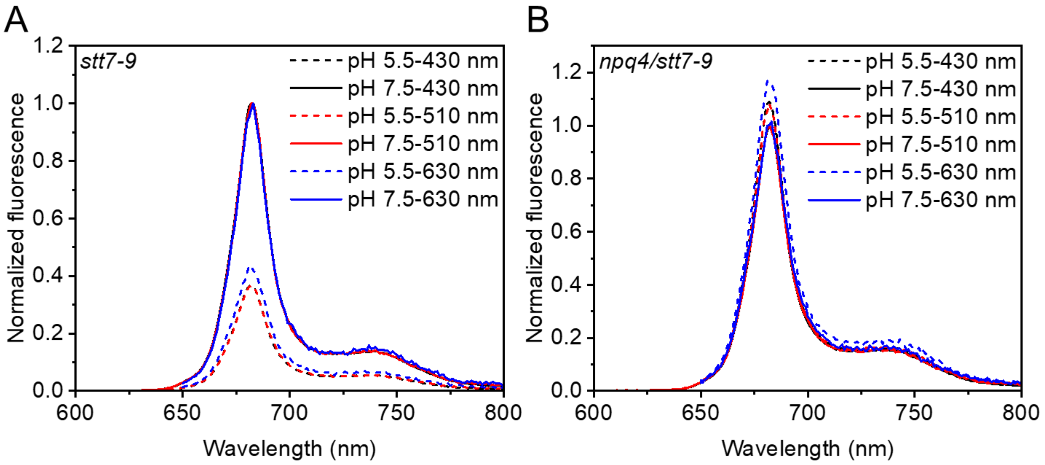

Supplementary Fig. 8. Room temperature fluorescence spectra of *stt7-9* (A) and *npq4/stt7-9* (B) micro-sized cell fractions at pH 5.5 and pH 7.5, samples were excited at 430, 510, and 630 nm, respectively. The spectra were normalized relative to the fluorescence maximum at pH 7.5. Source data are provided as a Source Data file.

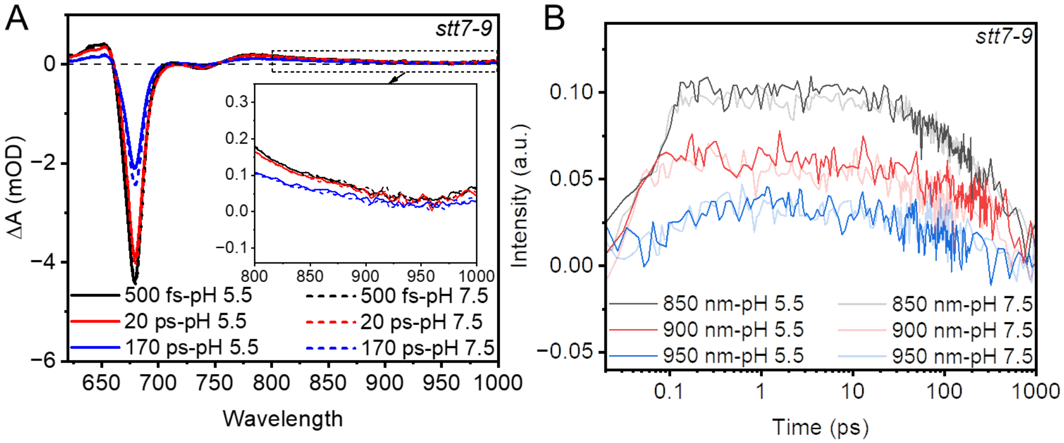

Supplementary Fig. 9. TA data of *stt7-9* micro-sized cell fractions in 620-1000 nm region. (A) The TA spectra at three different time-points of *stt7-9* samples at pH 5.5 and pH 7.5. Inset: The zoom-in spectra in the near-infrared region (800-1000 nm). (B) TA kinetics at 850, 900, and 950 nm of *stt7-9* samples at different pHs. The spectra and traces at different pHs were normalized to their bleaching maximum at around 680 nm at time zero. Source data are provided as a Source Data file.

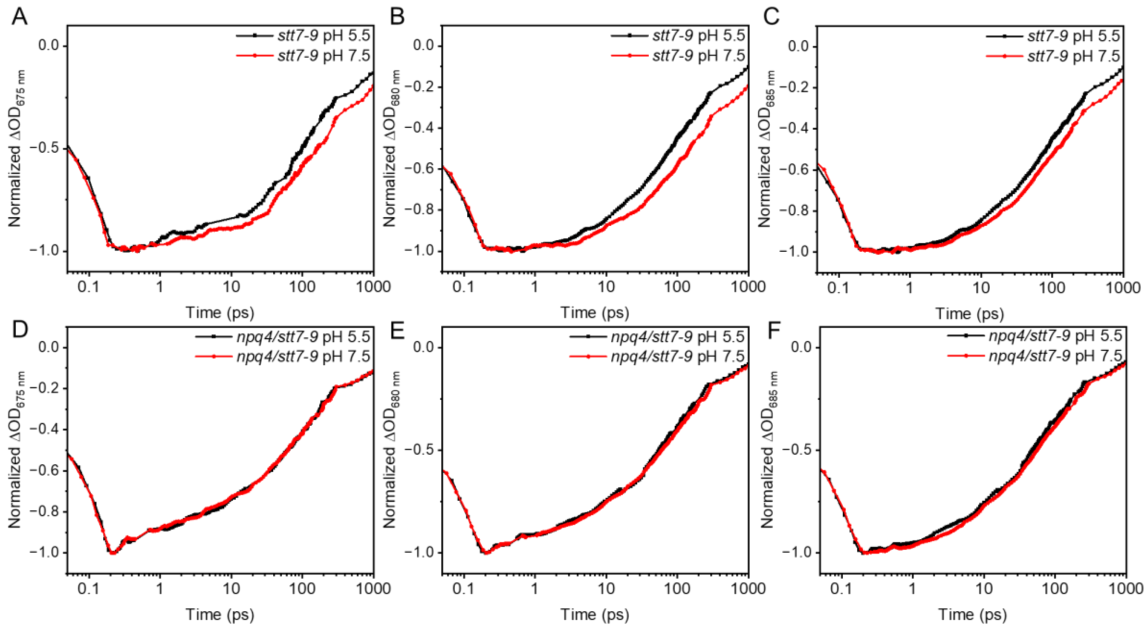

Supplementary Fig. 10. TA traces at 675, 680, and 685 nm for the *stt7-9* sample (A-C) and *npq4/stt7-9* sample (D-F) at different pHs. All the traces were normalized to the minimum. Source data are provided as a Source Data file.

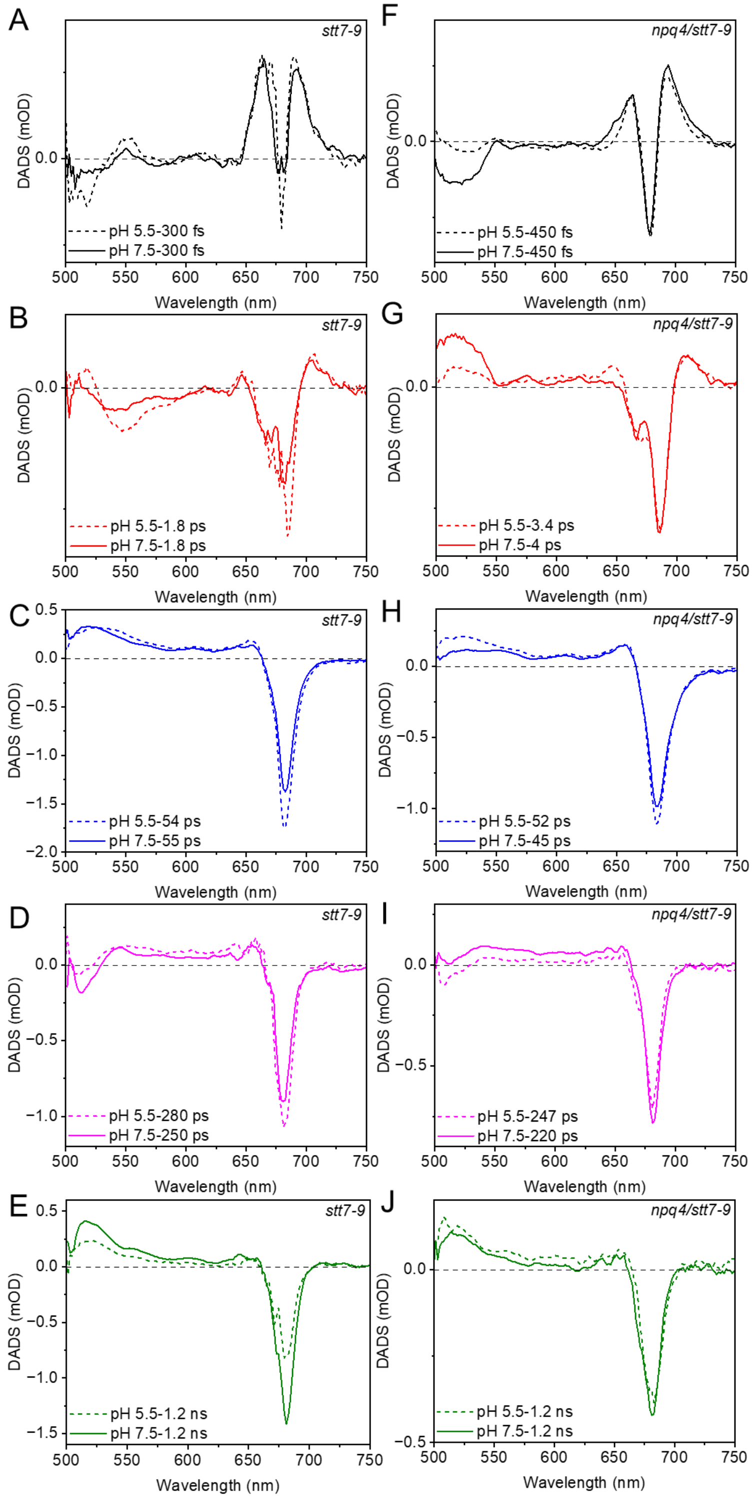

Supplementary Fig. 11. All individuals of DADSs of *stt7-9* (A-E) and *npq4/stt7-9* (F-J) samples were shown to highlight the differences induced by quenching. DADSs were normalized in the same way as shown in Figure 2. Source data are provided as a Source Data file.

Supplementary Table 1. The lifetime, assignment of the DADSs for *stt7-9* (Figure 2C), and the calculated proportion of the Chl-Q_y bleaching at 680 nm to the total at this wavelength.

***stt7-9* pH 7.5/*stt7-9* pH 5.5**

lifetime	assignment	The proportion of each DADS at 680 nm to the total Chl-Q_y bleaching
**55 ps/54 ps**	PSI, annihilation, and PSII quenching	37%/48%
**250 ps/280 ps**	PSII quenching and PSII_CS	25%/29%
**1.2 ns**	PSII_CS	38%/23%

EET: Excitation Energy Transfer; CS: Charge Separation

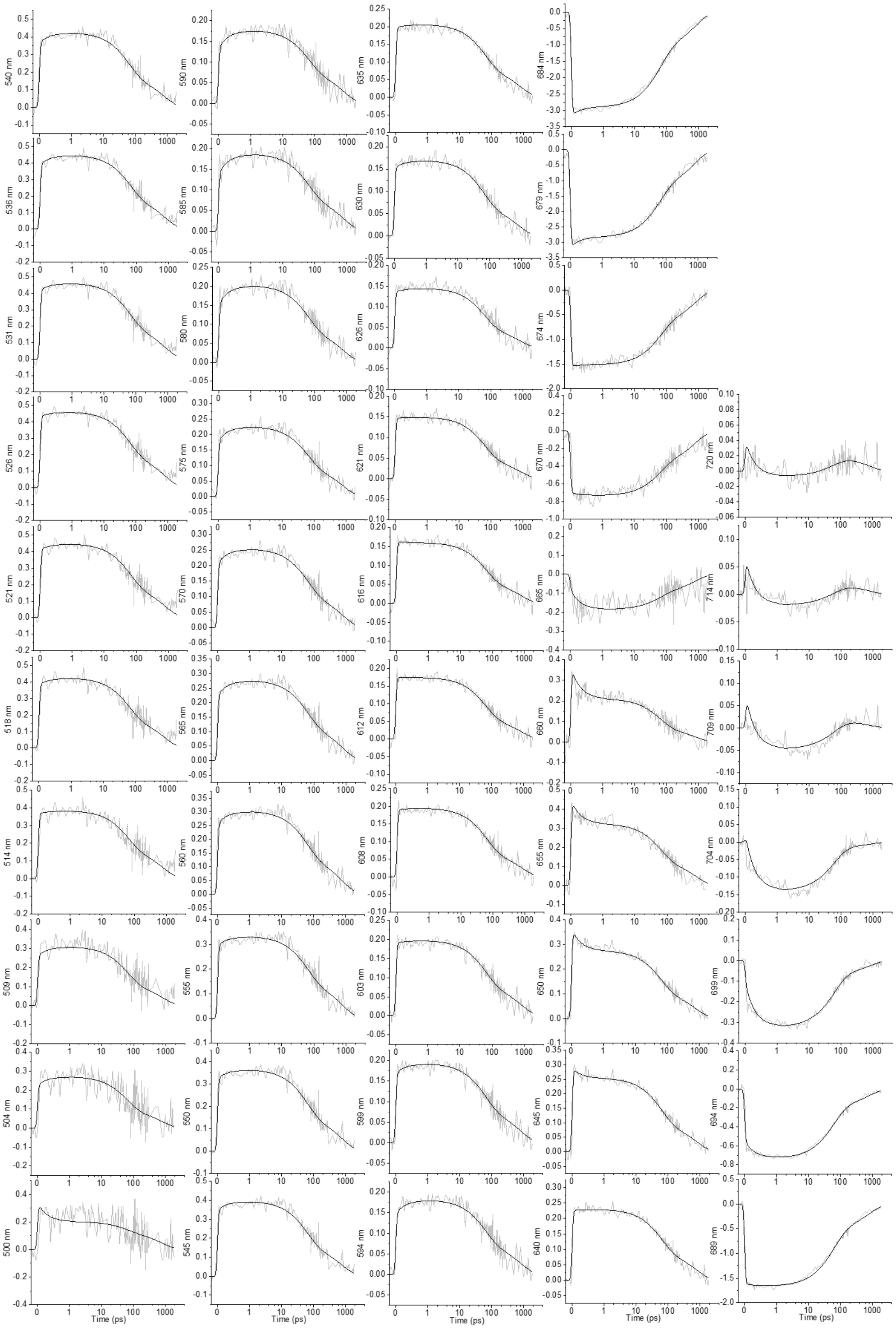

Supplementary Fig. 12. The time traces of transient absorption data (grey) and the fits (black) of *stt7-9* micro-sized cell fractions at pH 5.5 (related to Figure 2C). Wavelength is indicated in each panel, and the time scale is linear until 1 ps and logarithmic after that. Source data are provided as a Source Data file.

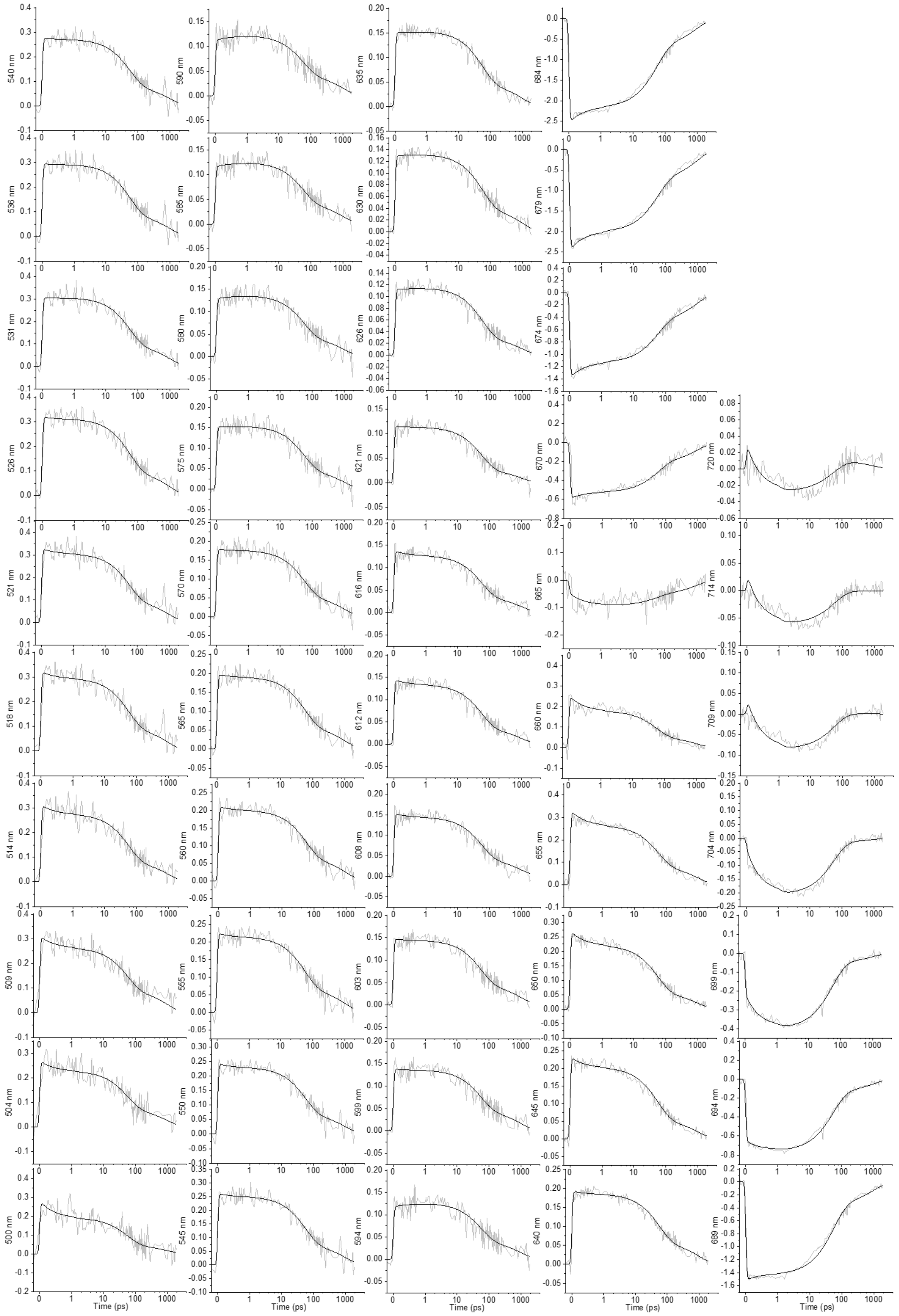

Supplementary Fig. 13. The time traces of transient absorption data (grey) and the fits (black) of *npq4/stt7-9* micro-sized cell fractions at pH 5.5 (related to Figure 2F). Source data are provided as a Source Data file.

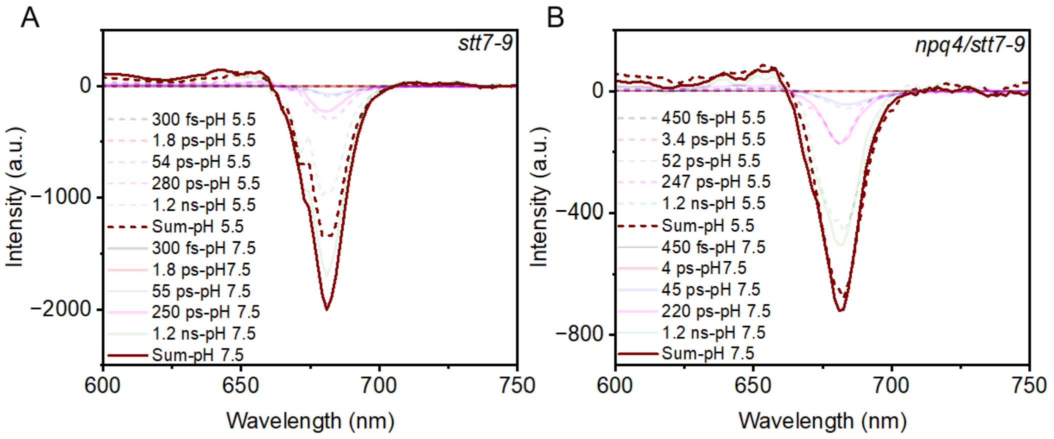

Supplementary Fig. 14. The steady-state fluorescence spectra were reconstructed from the DADSs of the two samples at different pHs (Figures 2C and F). The wine-dotted and solid lines represent the sum of the reconstructed spectra of each lifetime component of the sample at pH 5.5 and 7.5, respectively. Source data are provided as a Source Data file.

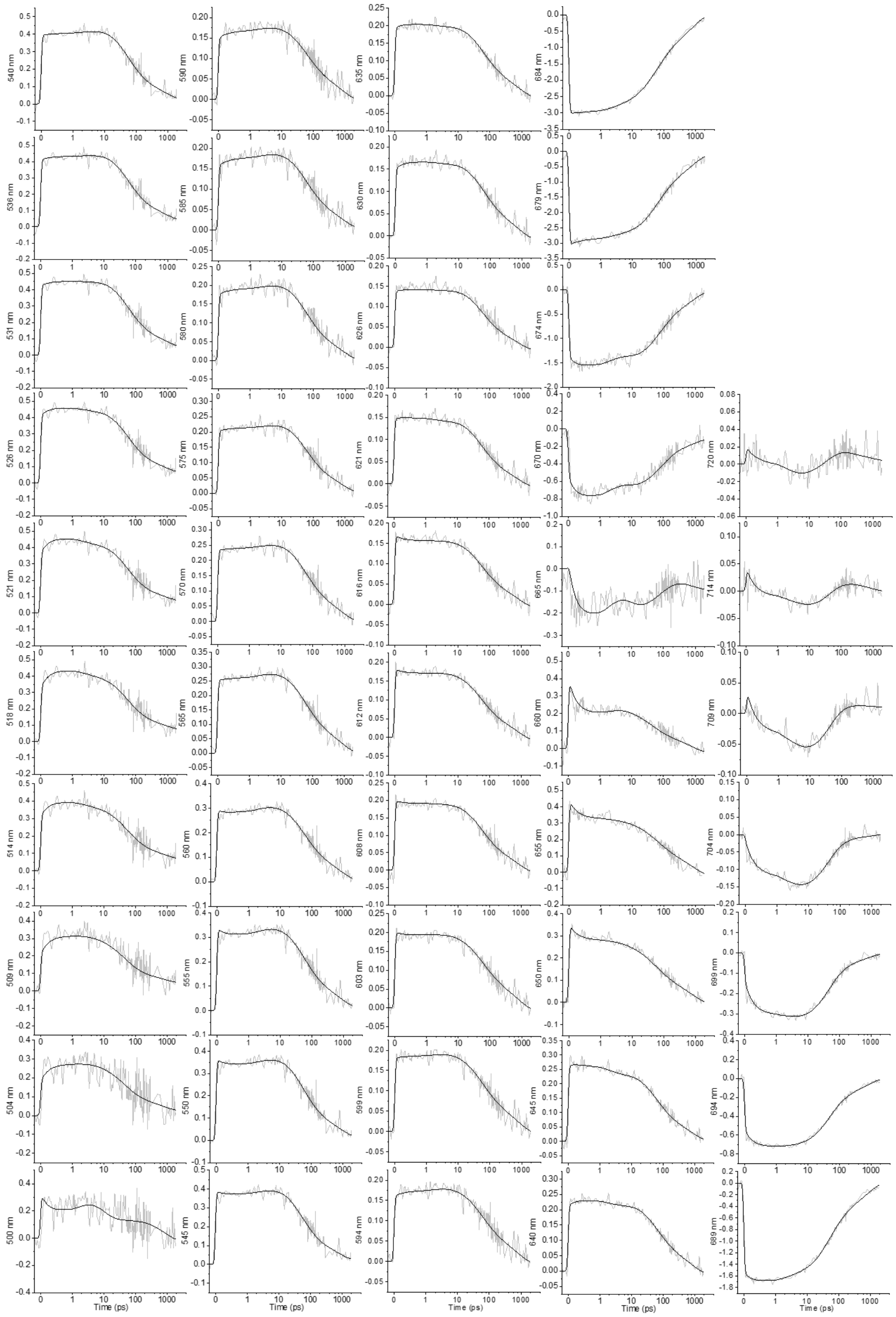

Supplementary Fig. 15. Transient absorption data and fitting (related to Figure 3B) of *stt7-9* micro-sized cell fractions at pH 5.5. The time traces of the raw data are plotted in grey (in mOD), and the fits are in black. Wavelength is indicated in the ordinate label of each panel, and the time scale is linear until 1 ps and logarithmic thereafter. Source data are provided as a Source Data file.

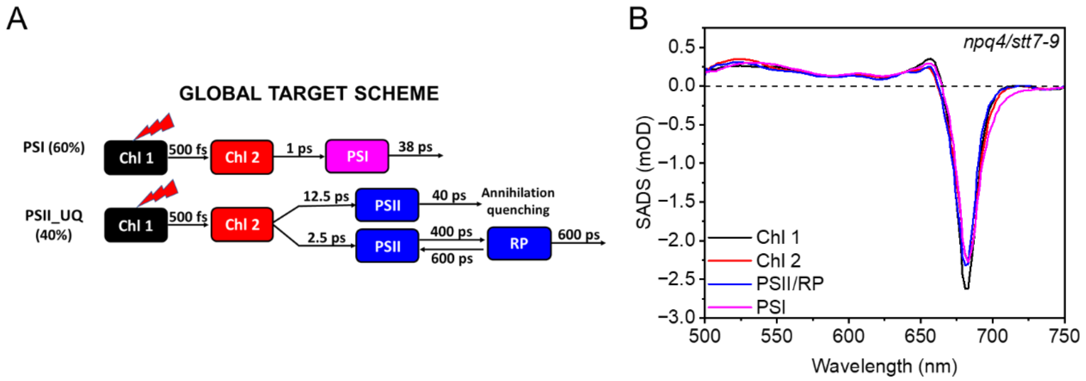

Supplementary Fig. 16. Global target analysis of TA data of *npq4/stt7-9* micro-sized cell fractions at pH 5.5 and pH 7.5. (A) The target model was used. (B) The obtained species-associated difference spectra (SADSs) of each compartment. Details about the constraints used in the model are explained in Supplementary Text 1. Source data are provided as a Source Data file.

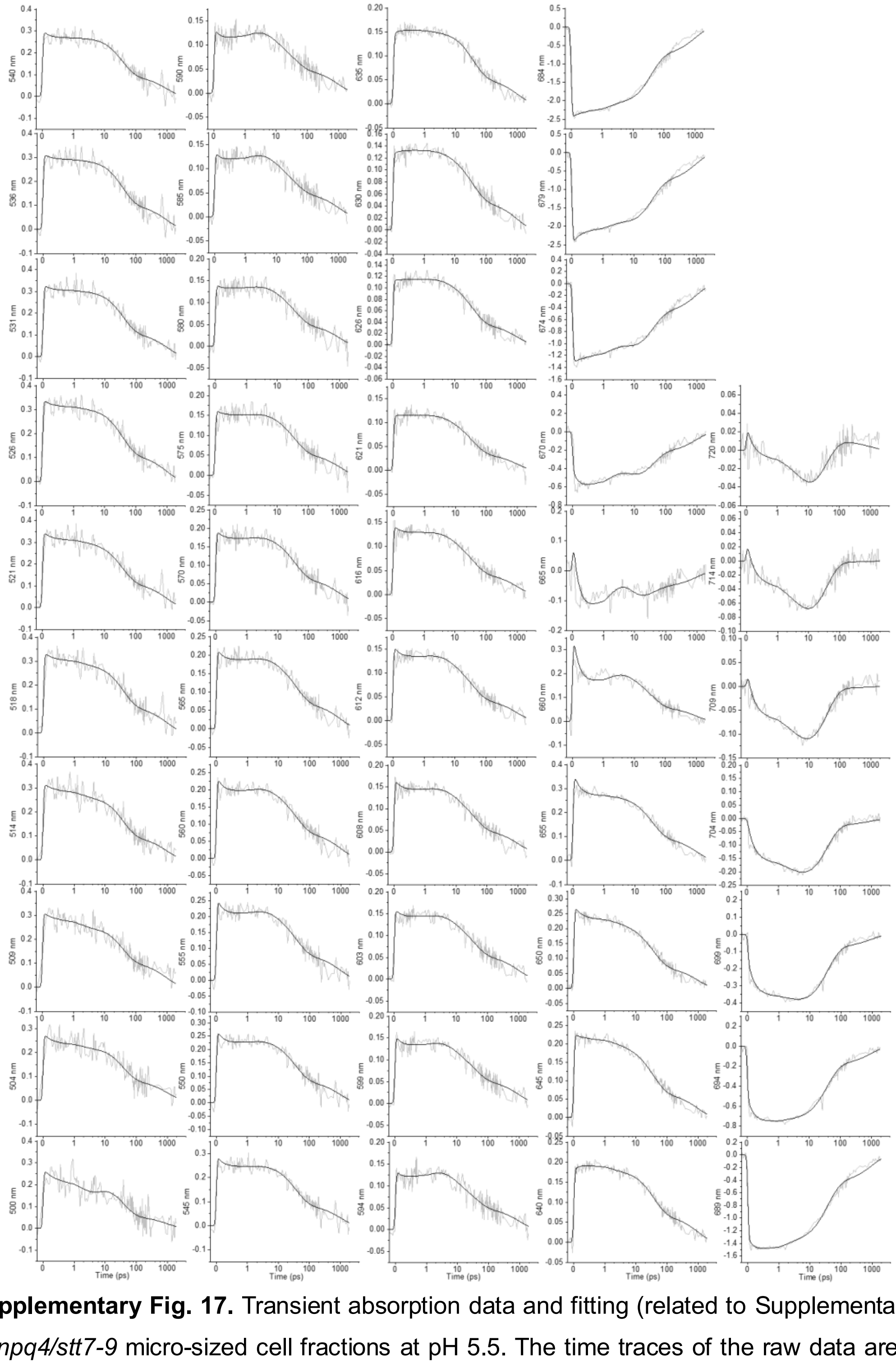

Supplementary Fig. 17. Transient absorption data and fitting (related to Supplementary Fig. 16) of *npq4/stt7-9* micro-sized cell fractions at pH 5.5. The time traces of the raw data are plotted in grey (in mOD), and the fits are in black. Wavelength is indicated in the ordinate label of each panel, and the time scale is linear until 1 ps and logarithmic beyond that. Source data are provided as a Source Data file.

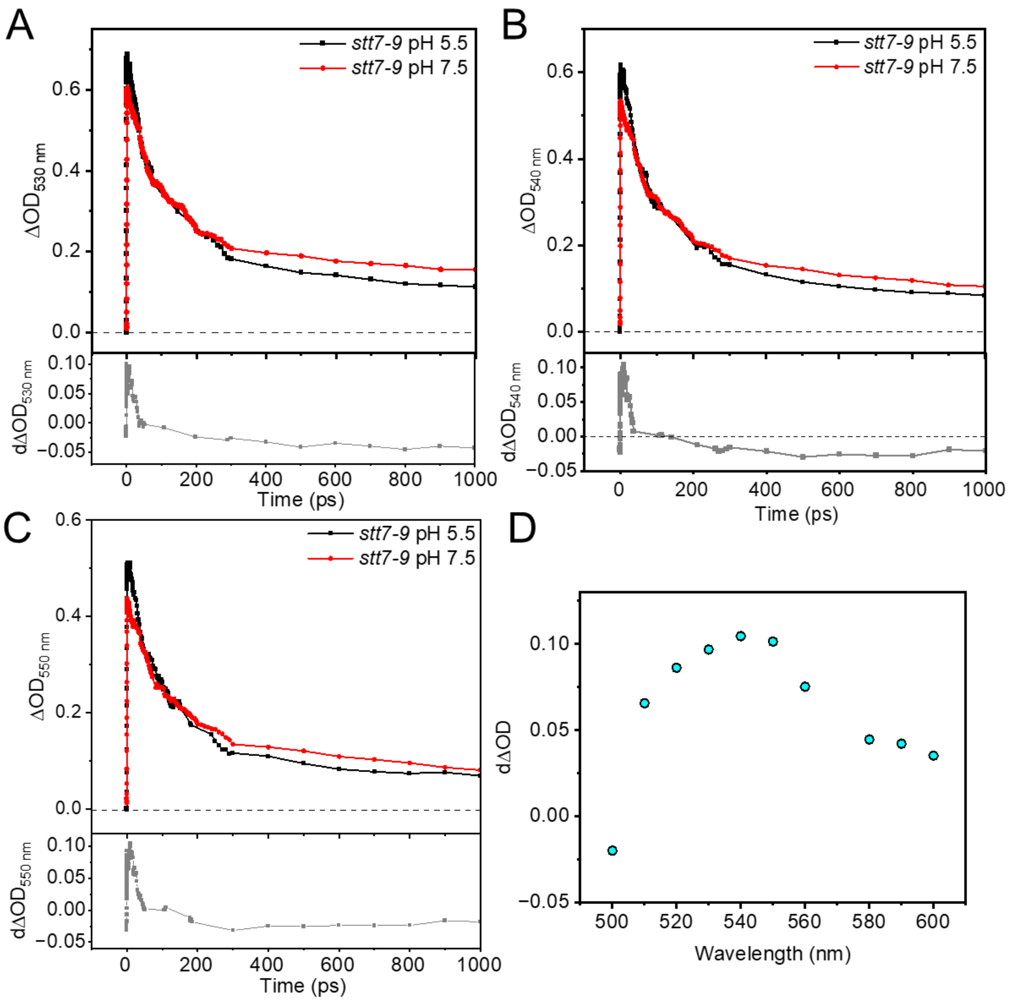

Supplementary Fig. 18. TA signals of *stt7-9* sample in the wavelength region of 500-600 nm. (A)-(C) The TA traces of *stt7-9* at 530, 540, and 550 nm (upper panel), and the decay traces were normalized at 50 ps, a time-point that the carotenoid ESA is largely disappearing and the Chl ESA is not yet quenching. The difference between unquenched (red) and quenched (black) curves (dΔOD) is shown by the gray trace (bottom panel). (D) The difference in ESA signal (dΔOD) in the 500-600 nm wavelength region of *stt7-9* sample was plotted against wavelength at 8 ps. Source data are provided as a Source Data file.

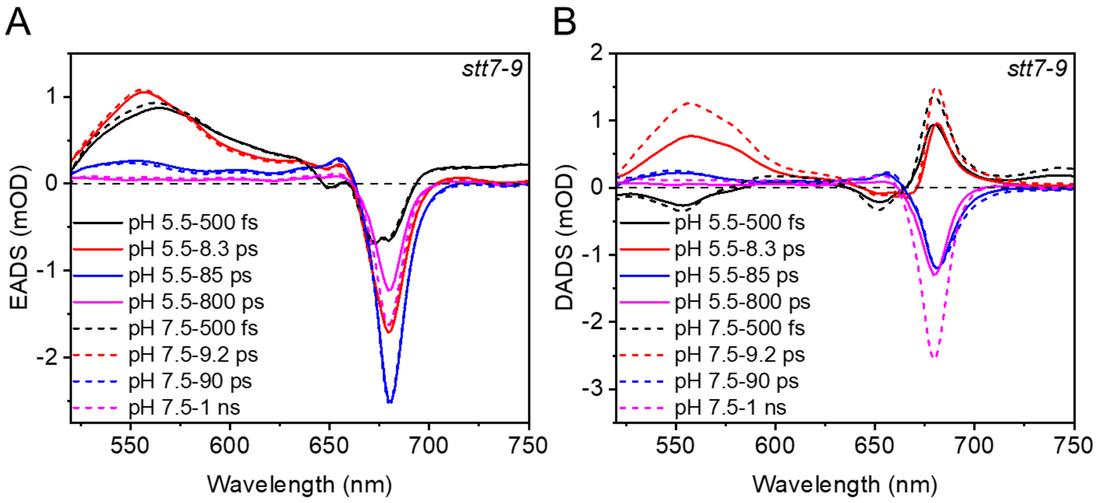

Supplementary Fig. 19. Transient absorption data of *stt7-9* micro-sized cell fractions at pH 5.5 and pH 7.5. EADS (A) and DADS (B) were obtained by global analysis of TA data upon direct carotenoid excitation (510 nm). The spectrum of the Car S_1 state was thus obtained, see the red DADS which decays to the ground state in 8.3 ps. Source data are provided as a Source Data file.

Supplementary Text 1. Details about the constraints used in the target analysis of *stt7-9* and *npq4/stt7-9*.

Two different experiments for each mutant of *stt7-9* and *npq4/stt7-9* were analyzed simultaneously in the global target analysis: unquenched (pH 7.5) and quenched (pH 5.5). Several constraints were applied in the fitting as listed below:

i) Spectral assumptions:

- Compartments in the same color are forced to have the same SADS (black, red, and blue compartments).
- The spectra of the triplets and quencher were forced to be zero in the ranges of 650-750 nm or 620-750 nm, respectively.

ii) Initial energy input parameters:

PSI, PSII, and Car triplet state can be kinetically distinguished by their lifetimes: PSI has a typical lifetime below 100 ps, while the second one usually decays in 200 ps to ns when its RC is closed and Car triplet state decays the slowest (>100 ns). Besides, PSI has a slightly red-shifted spectrum from PSII, making it even more distinguishable from other compartments. Note that, in our model, the fractions of excitation energy inputs on PSI and PSII determine the relative amplitudes of their SADS, which should be more or less equal as both originate from Chls. Therefore, through equalizing the minima of the Q_y bleaching signal of PSI (magenta compartment in Figure 3 and Supplementary Fig. 13) and PSII (blue compartment in Figure 3 and Supplementary Fig. 13), we determined that for *stt7-9* the relative excitation on PSII is about 65%, and for PSI 28% and the rest on Car triplet state (7%). In addition, the excitation energy inputs on PSII_Q in the stt7-9 model are roughly estimated according to the quenching proportion (~60%, see Figure 1C and Supplementary Fig. 7A) obtained in the steady-state spectra, making it ~39% (65% * 60%). The fraction of quenched PSII can be estimated in this way because the contribution from PSI (50 ps versus ns) to the steady-state spectra is neglectable.

To summarize, although all of the energy inputs were fixed during the fitting, these numbers were iteratively determined following the principle as explained above.

- For the mutant of *stt7-9*, at pH 5.5, four branches of PSII_quenched (PSII_Q), PSII_unquenched (PSII_UQ), PSI, and carotenoid triplet state account for 39% (60% of the total energy absorbed by PSII), 26% (40% of the total energy absorbed by PSII), 28% and 7% of the total initial excitation energy, respectively, whereas these fractions become 0%, 63%, 27% and 10% at pH 7.5.
- For the double mutant of *npq4/stt7-9*, the branch of PSII absorbs 40% of the initial

excitation energy, and PSI absorbs the other 60%.

iii) Lifetime parameters:

- The fastest energy transfer time of 250 fs and the triplet lifetime of 100 ns were both fixed.

References

1. Tian L, Nawrocki WJ, Liu X, Polukhina I, van Stokkum IHM, Croce R. pH dependence, kinetics and light-harvesting regulation of nonphotochemical quenching in *Chlamydomonas*. *Proc. Natl. Acad. Sci. U.S.A.* 2019, **116**(17)**:** 8320-8325.
2. Madireddi SK, Nama S, Devadasu ER, Subramanyam R. Photosynthetic membrane organization and role of state transition in *cyt*, *cpII*, *stt7* and *npq* mutants of *Chlamydomonas reinhardtii*. *J. Photochem. Photobiol. B, Biol.* 2014, **137:** 77-83.
